# miR-155-5p/Bmal1 Modulates the Senescence and Osteogenic Differentiation of Mouse BMSCs through the Hippo Signaling Pathway

**DOI:** 10.1007/s12015-023-10666-3

**Published:** 2023-12-27

**Authors:** Lanxin Zhang, Chengxiaoxue Zhang, Jiawen Zheng, Yuhong Wang, Xiaoyu Wei, Yuqing Yang, Qing Zhao

**Affiliations:** 1 Department of Orthodontics, State Key Laboratory of Oral Disease & National Clinical Research Center for Oral Diseases, West China School & Hospital of Stomatology, Sichuan University, 14, 3Rd Section of Ren Min Nan Rd, Chengdu, 610041 China; 2 Department of Stomatology, West China Fourth Hospital, Sichuan University, 18, 3Rd Section of Ren Min Nan Rd, Chengdu, 610041 China

**Keywords:** Bone Marrow Mesenchymal stem Cells, Bmal1, miR-155-5p, Hippo Signaling Pathway, Senescence, Osteogenesis

## Abstract

**Background:**

The core clock gene brain and muscle ARNT like-1 (Bmal1) is involved in the regulation of bone tissue aging. However, current studies are mostly limited to the establishment of the association between Bmal1 and bone senescence, without in-depth exploration of its main upstream and downstream regulatory mechanisms.

**Methods:**

The luciferase reporter assay, RT-qPCR and Western blotting were performed to detect the interaction between miR-155-5p and Bmal1. The effects of miR-155-5p and Bmal1 on the aging and osteogenic differentiation ability of mouse bone marrow mesenchymal stem cells (BMSCs) were investigated by cell counting kit-8 (CCK-8) assay, flow cytometry, β-gal staining, alkaline phosphatase quantitative assay and alizarin red staining in vitro. The potential molecular mechanism was identified by ChIP-Seq, RNA-seq database analysis and immunofluorescence staining.

**Results:**

The expression of Bmal1 declined with age, while the miR-155-5p was increased. miR-155-5p and Bmal1 repressed each other’s expression, and miR-155-5p targeted the Bmal1. Besides, miR-155-5p inhibited the proliferation and osteogenic differentiation of BMSCs, promoted cell apoptosis and senescence, inhibited the expression and nuclear translocation of YAP and TAZ. However, Bmal1 facilitated the osteogenic differentiation and suppressed the aging of BMSCs, meanwhile inactivated the Hippo pathway. Moreover, YAP inhibitors abrogated the positive regulation of aging and osteogenic differentiation in BMSCs by miR-155-5p and Bmal1.

**Conclusion:**

In mouse BMSCs, miR-155-5p and Bmal1 regulated the aging and osteogenic differentiation ability of BMSCs mainly through the Hippo signaling pathway. Our findings provide new insights for the interventions in bone aging.

**Graphical Abstract:**

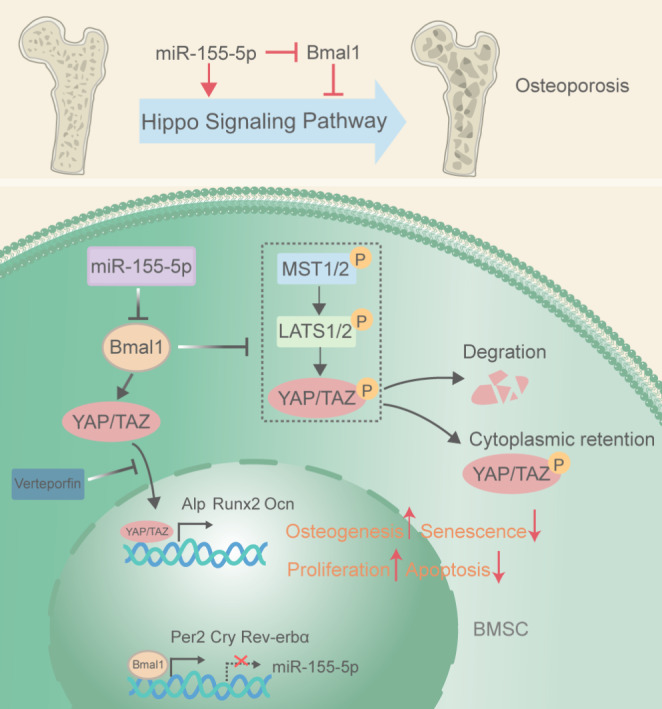

**Supplementary Information:**

The online version contains supplementary material available at 10.1007/s12015-023-10666-3.

## Introduction

With the extension of the human life span, senile osteoporosis has become a major chronic metabolic bone disease worldwide in recent years, characterized by decreased bone mass and increased bone fragility [1]. Bone loss during aging is mainly associated with reduced proliferation and osteogenic differentiation capacity, and the increased adipogenesis of bone marrow mesenchymal stem cells (BMSCs) [2, 3]. Furthermore, senescent BMSCs display a decreased migratory capacity and therapeutic immunoregulatory activity, secrete higher levels of proinflammatory chemokines such as IL6, IL8, MCP1 to exhibit a senescence-associated secretory phenotype (SASP), which expand the number of senescent cells and even promote the cancer initiation [4]. The aging process of BMSCs involves alterations in metabolism, multiple signaling pathways, genetics and epigenetic regulation, and the complex molecular regulatory mechanisms still need to be further explored [5].

Body temperature, hormone levels and behavioral patterns show daily rhythms, and the disturbed rhythms are closely related to neurodegenerative, metabolic diseases and premature aging. Both central and peripheral circadian rhythms are regulated by core clock genes, including Bmal1, Per, Clock, and Cry, among which Bmal1 is indispensable to the positive regulatory rhythm system [6]. Growing evidence suggests that Bmal1 plays an important regulatory role in bone aging [7, 8]. Bmal1^-/-^ mice showed accelerated aging, shortened lifespan, reduced hair, increased bone resorption and a low bone mass phenotype [8, 9]. Overexpression of Bmal1 promoted the proliferation and osteogenic differentiation of BMSCs, while inhibition diminished bone formation and promoted bone resorption activity of osteoclasts [10–12]. However, most of the current studies have not explored the mechanism in-depth, and the results are controversial. It has been suggested that Bmal1 regulated BMSCs through the BMP2 pathway [13], while others found the Wnt or P53 pathways [14, 15]. However, the main core pathway by which Bmal1 regulates the osteogenic differentiation capacity of BMSCs has not been verified.

MiRNAs are small single-stranded noncoding RNAs with a length of 19–24 nucleotides. They are involved in more than 90% of physiological and pathological processes in vivo, including the bone aging [16, 17]. The expression of miRNAs varies with age, and some regulate the clock genes such as Bmal1 and Clock at post-transcriptional levels [18, 19]. Is the age-related decline in Bmal1 expression in bone tissue regulated by miRNA? We screened potential miRNAs targeting Bmal1 by the following target prediction software: Pictar, TargetScan and miRanda. The intersection of miRNAs with high scores was calculated, and the results showed that miR-155-5p had the strongest association with Bmal1. Moreover, previous studies have found that miR-155-5p reduces the expression levels of Bmal1 [20]. However, the expression of miRNA is tissue specific [21]. Can miR-155-5p target Bmal1 in BMSCs? What kind of feedback regulation do they have? How does crosstalk affect the aging of BMSCs? What is the regulatory mechanism?

The Hippo signaling pathway has been widely studied in cell proliferation, cell development, and cancer. Recently, some studies have indicated that the Hippo pathway plays a vital role in the regulation of cell differentiation in cells such as BMSCs [22–24]. The downstream effectors of Hippo pathway, YAP and TAZ, were reported to positively regulate osteogenic differentiation [25], and TAZ inhibited the transcription mediated by PPARγ [26]. The Hippo signaling pathway also interacts with other osteogenesis-related pathways such as the Wnt/β-catenin, BMP2, and Hedgehog pathways, and their impact on osteogenic differentiation involves the participation of the Hippo. Bmal1 has recently been stated to regulate the proliferation and self-renewal of intestinal epithelial cells through the Hippo pathway. The upregulation of YAP1 promotes the transformation of smooth muscle cells into fibroblasts suggesting that the Hippo pathway may be involved in the regulation of stem cell function and rhythm downstream of Bmal1 [27].

Based on the above studies, we proposed that miR-155-5p could target the regulation of Bmal1, and their interaction could modulate the aging and osteogenic differentiation capacity of BMSCs. Here, we found that the expression trends of miR-155-5p and Bmal1 in young and aged mouse BMSCs were opposite, with Bmal1 showing an age-related reduction and miR-155-5p showing an age-related increase in expression. Furthermore, we provide evidence that miR-155-5p and Bmal1 can inhibit each other, and that miR-155-5p targets the 3’UTR of Bmal1. In addition, miR-155-5p also inhibits the expression of other core clock genes, such as Cry, Per2 and Rev-erbα. The ChIP-Seq and RNA-seq database analysis of the BMAL1 protein showed that it was closely related to the Hippo pathway. Noticeably, the in vitro studies showed that miR-155-5p and Bmal1 could both mediate the proliferation, apoptosis, senescence, and osteogenic differentiation of BMSCs by orchestrating the nuclear translocation of YAP and TAZ, the Hippo pathway downstream effecting factors, and YAP inhibitors could block their effects on BMSCs.

## Materials and Methods

### Isolation and Culture of BMSCs

BMSCs were derived from C57/BL6 male mice purchased from Chengdu Dossy Experimental Animals Company (China). Young mice were 1 month of age and old mice were 12 months of age. After mice were sacrificed by neck dislocation, the muscles were stripped, and the femur and tibia were separated. The bone marrow cavity was rinsed with complete medium containing 9% fetal bovine serum (FBS) (Gibco, USA), 90% α-MEM medium (Gibco, USA), and 1% penicillin-streptomycin (Hyclone, USA). After being blown into a single cell suspension, the cells were uniformly planted into a 25cm^2^ culture flask and incubated at 95% relative humidity, 37℃ and 5% CO_2_. The medium was changed every two days. P3 cells were characterized by the morphology of spindle-shaped, growing in a swirling arrangement and plastic-adherent way as observed under the inverted microscope, which were used in all experiments.

Cells were seeded in 6-well plates at 1 × 10^5^ cells per well for the induction of osteogenic and adipogenic differentiation. The osteogenic differentiation induction medium contained 10% FBS, 50 µg/ml vitamin C, 10 mmol/L β-sodium glycerophosphate, and 10 nmol/L dexamethasone (Sigma, USA). The adipogenic induction medium contained 10% FBS, 1ug/ml insulin, 1 μm dexamethasone, and 0.5mM methyl-isobutylxanthine. To detect osteogenic differentiation ability, quantitative determination of alkaline phosphatase activity was performed 7 days after osteogenic induction (Beyotime, China), and alizarin red staining was performed 7 and 14 days after osteogenic induction (Cyagen Biosciences, China). The adipogenic differentiation ability was examined by Oil Red O staining after the two weeks of adipogenic induction (Beyotime, China).

### Flow Cytometry Analysis

Flow cytometry was used for cell phenotype identification and apoptosis rate determination. The collected cells in each group were washed with PBS three times at 1000 rmp/min for 3 min, followed by resuspension in 100 µl of PBS. Five microliters of CD29-FITC (Abcam, USA) and 5 µl of CD34-Phycoerythrin (Abcam, USA) were added to each tube, and the cells were incubated for 30 min in the dark. Cell surface antigens were identified by the AttuneNxt (Thermo, USA) flow sorter after 1000 µl of PBS supplementation. For apoptosis rate detection, the density of the cell suspension was adjusted to 1 × 10^6^ cells/ml. After washing with PBS, 70% ethanol was added to the cells, and the cells were fixed at 4℃ for 2 h. After filtering through a 400-mesh screen, 1 mmol/L JC-1 was added for staining, and the cells were stained at 37℃ for 30 min (shielded from light). Finally, the apoptosis of each group was determined by flow cytometry.

### Real-time Quantitative PCR Analysis (RT-qPCR)

An RNA rapid extraction kit (ES Science, China) was used to extract RNA from BMSCs. Reverse transcription for mRNA was performed using the Prime Script RT Reagent Kit with gDNA Eraser (Takara, Japan), while the miRNA First Strand cDNA Synthesis (Sangon Biotech, China) kit was utilized for miR-155-5p. PCR chain reaction was performed with reference to the TB Green Premix Ex TaqTM II (Takara, Japan) kit. β-Actin was used as the internal reference, and U6 was used for miRNA. All experiments were repeated three times, and the relative mRNA expression levels of all groups were expressed as 2^−△△Ct^. The primer sequences used in the study were listed in supplementary Table 1.

### Cell Transfection

BMSCs were plated on 6-well plates at 5 × 10^5^ cells per well, and virus transfection was performed untill the cells reached 50% confluence. The amount of virus required by each well cell was added according to the optimal multiplicity of infection (MOI) value (MOI = 20–30) with 1 ml of medium. After 4 h of cultivation, the medium was supplemented to 2 ml. After 24 h of virus transfection, the medium was replaced with fresh medium. The green fluorescence was observed under fluorescence microscope after 48 h. The preparation, packaging, and quality inspection of lentivirus vectors, carrying overexpressed miR-155-5p (miR-155-5p-OE), scrambled control (Scrambled), sponged miR-155-5p (miR-155-5p-sponge), mock control (Mock), overexpressed Bmal1 (Bmal1-OE), control of Bmal1-OE (Bmal1-OE-Ctrl), shRNA targeting Bmal1 (sh-Bmal1), control of sh-Bmal1 (sh-Bmal1-Ctrl) respectively, were completed by Hanbio Biotechnology (Shanghai). The non-treated BMSCs were used as blank control group (Ctrl).

### Western Blotting Assay

Total cell protein was extracted from BMSCs according to the SAB Total Protein Extraction Kit (SAB, USA) kit instructions from BMSCs. A BCA (Beyotime, China) assay kit was used to measure the protein concentration. 30 micrograms of protein from each sample was subjected to sodium dodecyl sulphate-polyacrylamide gel electrophoresis (SDS-PAGE), and then transferred to polyvinylidene fluoride (PVDF) membranes. After blocking with skim milk, the membranes were incubated with the following primary antibodies overnight at 4℃: Bmal1 (Abcam, ab93806), GAPDH (CST, 2118 S), YAP (Abcam, ab205270), p-YAP (Abcam, ab76252), TAZ (Abcam, ab84927), MST1 (CST, 3682 S), MST2 (CST, 3952 S), P-MST1/2 (CST, 49,332 S), LATS1 (Abcam, ab70561), LATS2 (Abcam, ab135794), ALP (Affinity, DF12525), RUNX2 (Bioss, bs-1134R), OCN (Affinity, DF12303), P53 (Abcam, ab26), P16 (Abcam, ab80) and β-actin (Abmart, P30002). The antibodies were diluted in Tris-buffered saline (TBS) with Tween-20 (TBST) at 1:1000. Subsequently, the membranes were immersed in HRP universal secondary antibody (1:2000, CST, 7074,) for 1 h. Afterwards, the protein bands were visualized according to the ECL kit (Millipore, USA), and the gray values were analyzed using ImageJ software.

### Dual-luciferase Assay

The possible target sites of miR-155-5p on Bmal1 mRNA were predicted using the TargetScan, miRanda and miRDB databases. The 3’UTR and mutant sequence of Bmal1 were cloned in the Ps-Check2 vector (Promega, USA) to construct a luciferase reporter vector. After transfection with pSI-Check2-wt-Bmal1 or pSI-Check2-mut-Bmal1 recombinant plasmids, BMSCs were incubated with miR-155-5p mimics or miR-155-5p mimic NC for 24 h. Then, the Dual-Luciferase Reporter Assay System kit (Promega, USA) was used to measure the luciferase activity after another 24 h of cultivation in complete medium.

### Cell Proliferation Assay

Cell suspensions were prepared for each group of cells, and then seeded into 96-well plates at 1 × 10^4^ cells per well. Ten microliters of CCK-8 solution was added to each well (Beijing Renhua, China) for 4 h after 1, 3, 5, 6 and 7 days of incubation at 37℃. Finally, the absorbance at 450 nm was measured by an enzyme marker (Thermo Fisher, USA).

### β-Galactosidase(β-gal) Staining

Cells were plated in 6-well plates at 1 × 10^5^ cells per well and fixed at room temperature for 15 min using 1 ml of staining fixative (Solarbio, China). One milliliter of working solution was added to each well, and then the cells were incubated overnight at 37℃. The blue-stained cells were observed and counted under a phase-contrast microscope. The positive staining rate was determined by dividing the number of blue-stained cells with the total number of cells observed.

### ChIP-Seq and Data Analysis

BMSCs were fixed with formaldehyde, and DNA was cross-linked with protein. The chromatin was cleaved by ultrasonic fragmentation after lysis of the cells, and the DNA was purified from the immune complex by overnight precipitation with anti-Bmal1 antibody (CST, USA) at 4 ℃. Illumina HiSeq was used to sequence the library, and FastQC was used to control and screen the original data. Burrows Wheeler Aligner (BWA) was used to align the read segment with the reference sequence, and MACS2 software was applied to complete the peak detection analysis. MEME and Dreme software were used to detect significant motif sequences in peak sequences, and Tomtom software used the known motif database for annotation. GO function and KEGG enrichment analyses were performed for the genes with peaks.

### Immunofluorescence Staining

The BMSCs were fixed in paraformaldehyde for 30 min. Then, 0.2% Triton X-100 was used for permeation at room temperature for 20 min and the cells were rinsed 3 times with PBS. The samples were blocked with 5% sheep serum at room temperature for 60 min and incubated with primary antibodies against YAP (1:300; Abcam, ab205270) and TAZ (1:250; Abcam, ab84927) at 4℃ overnight. Subsequently, Cy3-labeled secondary antibody (1:1000; KPL, 072011506) was used for 2 h of incubation at room temperature avoiding light. Then, 0.01 mol/L of PBST was used for 4 times of rinsing away from light. The images were observed under a fluorescence microscope after the 50 µl of anti-quenching agent containing DAPI was added.

### Statistical Analysis

The obtained data are presented as the mean ± standard deviation (mean ± SD), and statistical analysis was performed with SPSS software (SPSS Statistics 23.0, IBM, USA). Graphing was performed using GraphPad (GraphPad Prism 8.4, GraphPad Software USA). One-way analysis of variance (ANOVA) and LSD post hoc test were used to compare the differences between the groups. The differences were considered statistically significant when *P* < 0.05.

## Results

### The Expression Trend of miR-155-5p and Bmal1 in the BMSCs of Young and Old Mice was Opposite

**Fig. 1:**
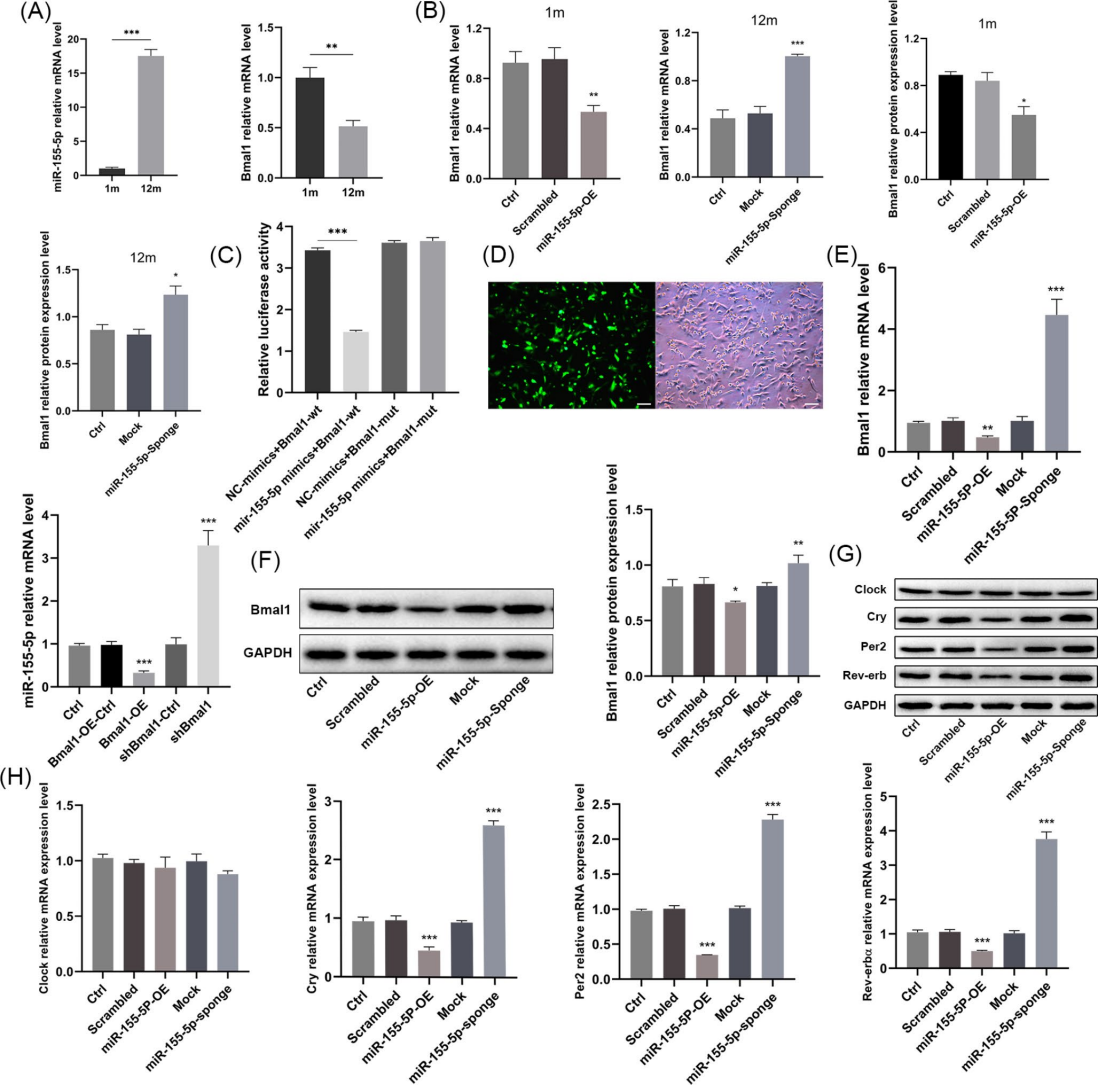
The interaction between miR-155-5p and Bmal1 and the effects of miR-155-5p on other circadian clock genes. (**A**) Relative background mRNA levels of miR-155-5p and Bmal1 in BMSCs of young (1 m) and aged (12 m) mice were determined by RT-qPCR. (**B**) mRNA and protein expression of Bmal1 were examined by RT-qPCR and Western blotting after inhibition or overexpression of miR-155-5p by lentivirus. (**C**) Relative fluorescence activity of BMSCs after transfection of miR-155-5p mimics or negative control. (**D**) Representative pictures of the fluorescence field and bright field of BMSCs after virus transfection, scale bar = 100 μm. (**E**) The effect of miR-155-5p and Bmal1 on their mutual expression level was detected by RT-qPCR. (**F**) The effect of miR-155-5p on the protein level of Bmal1 was determined by Western blotting. (**G**) Effects of miR-155-5p on the protein levels of Clock, Cry, Per2 and Rev-erbα; (**H**) Effects of miR-155-5p on the mRNA levels of Clock, Cry, Per2 and Rev-erbα. The data are shown as the mean ± SD, n = 3. *P < 0.05, **P < 0.01, ***P < 0.001

The flow cytometry analysis showed that the BMSCs expressed CD29 but lack of CD34 (Supplementary Fig. 1a). The osteogenic and adipogenic differentiation ability were identified by the formation of mineralized nodules and lipid droplets (Supplementary Fig. 1b). The mRNA expression of miR-155-5p in the BMSCs of aged mice was higher than that in the young mice, whereas the expression of Bmal1 was declined with aging (Fig. 1a). To further ask whether the miR-155-5p affects the level of Bmal1, we controlled the miR-155-5p using lentivirus in BMSCs. The results showed that the miR-155-5p overexpression decreased the mRNA and protein expression of Bmal1 in the young group and the miR-155-5p inhibition elevated in the old group (Fig. 1b, Supplementary Fig. 1c, d).

### miR-155-5p Targeted and Had a Reciprocal Suppressive Effect with Bmal1, Simultaneously Modulating Other Core Clock Genes

The targeting relationship between miR-155-5p and Bmal1 was suggested by analysis by TargetScan, miRanda and other databases, and the results showed that the target region was located at bases 40–47 and 235–241 of the Bmal1 3’UTR (Supplementary Fig. 1e). The luciferase reporter assay showed that miR-155-5p mimics markedly attenuated the fluorescence of BMSCs transfected with the pSI-Check2-wt-Bmal1 plasmid, while there was no difference in luciferase activity in the mut-Bmal1 group compared with the negative control, indicating that miR-155-5p was able to bind to the 3’UTR of Bmal1 (Fig. 1c).

Then, the miR-155-5p/Bmal1 overexpression/inhibition stable BMSC cell lines were generated to ascertain the interaction between miR-155-5p and Bmal1. (Fig. 1d, Supplementary Fig. 1f, g). We found that overexpression of miR-155-5p blocked the expression level of Bmal1, whereas silencing of miR-155-5p promoted it. Meanwhile, overexpression of Bmal1 also decreased miR-155-5p level, suggesting a mutual suppression between miR-155-5p and Bmal1(Fig. 1e, f). Next, the effects of miR-155-5p on other core Clock genes such as clock, Cry, Per2 and Rev-erbα, were tested. The results suggested that miR-155-5p presented no significant effect on the mRNA expression of Clock, but the effect on Cry, Per2 and Rev-erbα showed the same trend as Bmal1. Collectively, overexpression of miR-155-5p reduced the transcription levels of Cry, Per2 and Rev-erbα, while inhibition of miR-155-5p facilitated their expression (Fig. 1g, h).

### miR-155-5p and Bmal1 Mediated the Aging and Osteogenic Differentiation of BMSCs

**Fig. 2:**
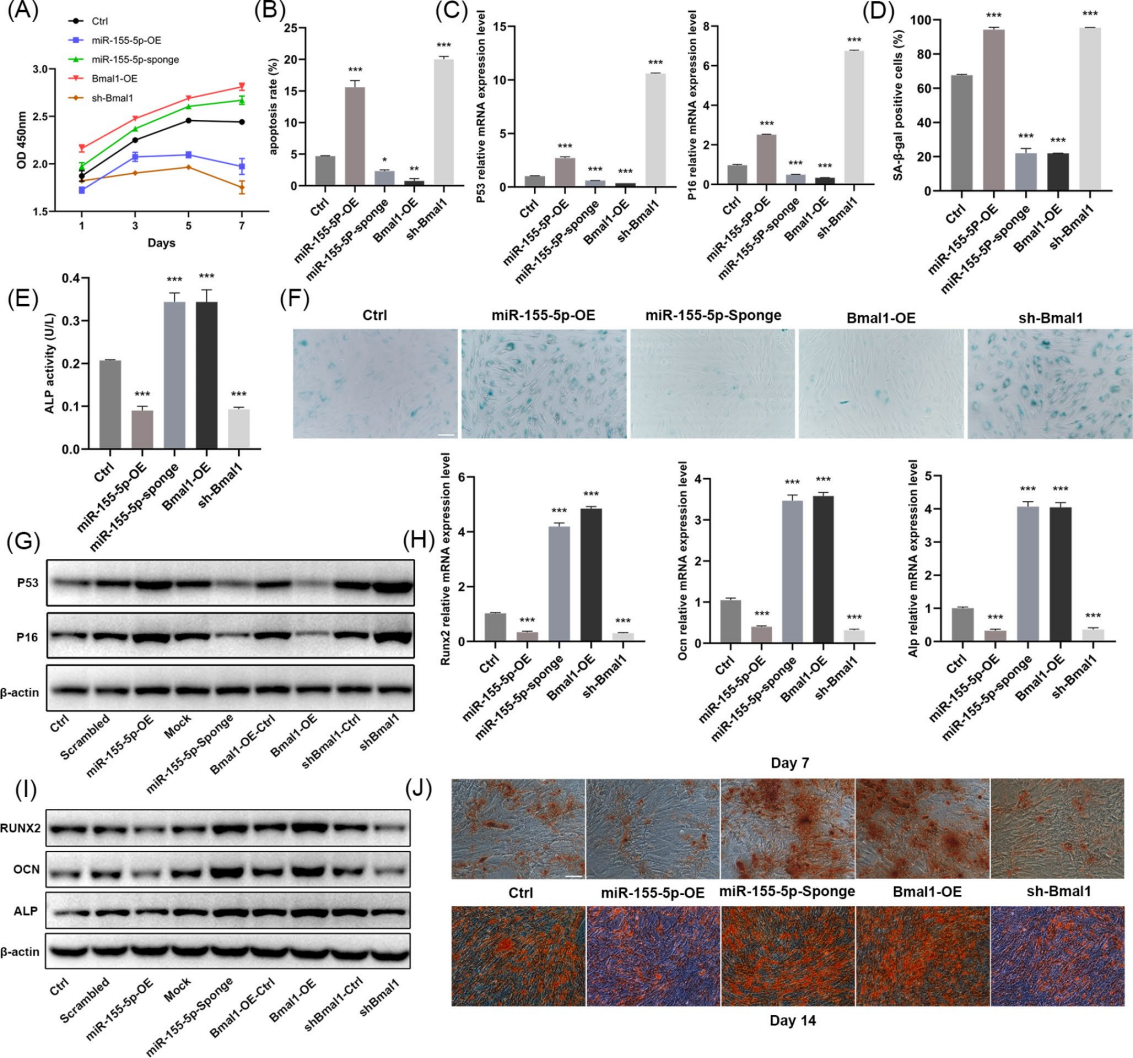
Effects of miR-155-5p and Bmal1 on the aging and osteogenic differentiation ability of mouse BMSCs. (**A**) Proliferation of BMSCs in different treatment groups was analyzed by CCK-8. (**B**) The effects of miR-155-5p and Bmal1 on the apoptosis ratio were detected by flow cytometry. (**C**) RT-qPCR analysis estimating the mRNA levels of P53 and P16 in different groups. (**D**) Percentage of β-gal-positive staining cells in different groups. (**E**) ALP activity of BMSCs in different treatment groups after 7 d of osteogenic induction. (**F**) Representative images of senescent BMSCs in different groups stained by β-gal, scale bar = 200 μm. (**G**) Western blotting analysis of P53 and P16 in different groups. (**H**) Relative mRNA expression levels of Runx2, Ocn and Alp in different treatment groups were investigated by RT-qPCR after 7 d of osteogenic induction. (**I**) Western blotting for Runx2, Ocn, Alp in different treatment groups after 7 d of osteogenic induction. (**J**) Alizarin red staining for mineralized nodules in different treatment groups after 7 d and 14 d of osteogenic induction respectively, scale bar = 200 μm. The data are shown as the mean ± SD, n = 3. *P < 0.05, **P < 0.01, ***P < 0.001

The CCK-8 assay demonstrated that miR-155-5p overexpression and Bmal1 interference largely reduced the proliferative activity of BMSCs, whereas miR-155-5p interference and Bmal1 overexpression groups both accelerated proliferation (Fig. 2a). Consistently, miR-155-5p overexpression/Bmal1 interference elevated the cell apoptosis rate and the mRNA and protein expression of P53 and P16, as well as significantly increasing the proportion of senescent BMSCs. In contrast, miR-155-5p interference/Bmal1 overexpression had the opposite effect and reduced the above age-related indicators (Fig. 2b-g).

**Fig. 3:**
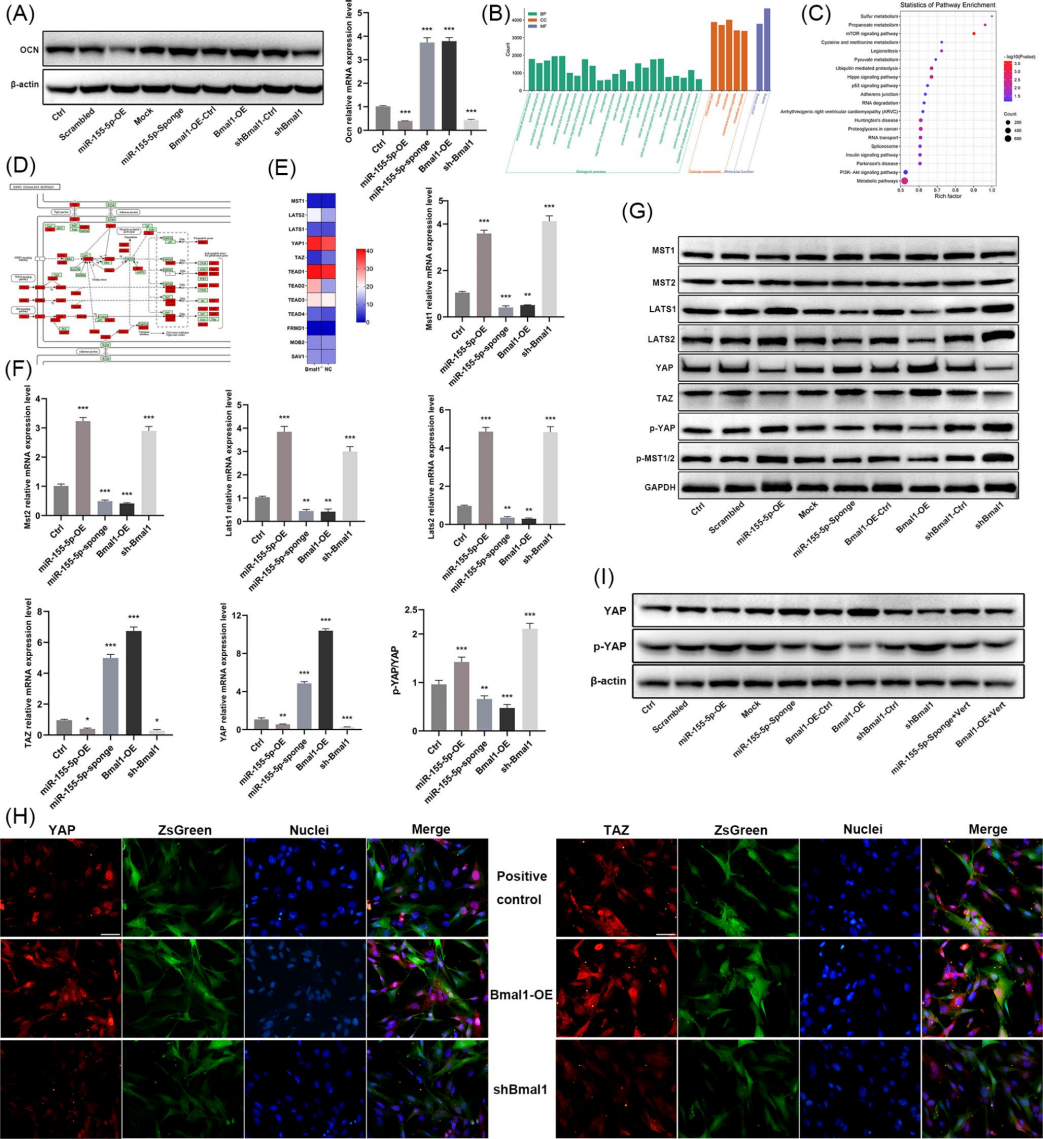
miR-155-5p and Bmal1 regulated the activation of the Hippo signaling pathway in mouse BMSCs. (**A**) Western blotting and RT-qPCR analysis of Ocn after 14 days of osteogenic induction. (**B**) GO enrichment analysis showing the biological processes involved by differentially expressed genes. (**C**) KEGG pathway enrichment analysis showed the 20 pathways with the most enriched differentially expressed genes. (**D**) The differentially expressed genes enriched in the Hippo pathway. (**E**) Heatmap of representative genes of the Hippo pathway in Bmal1^−/−^ and wild-type mouse BMSCs (GenBank ID: GSE208729). (**F**) RT-qPCR analysis of Mst1, Mst2, Lats1, Lats2, Yap and Taz in different groups of BMSCs, and the relative protein expression ratio of p-YAP and YAP. (**G**) Western blotting for Mst1, Mst2, Lats1, Lats2, YAP and TAZ protein levels in all groups. (**H**) Immunofluorescence staining for YAP and TAZ in BMSCs of the Bmal1 overexpression and Bmal1 inhibition groups, scale bar = 50 μm. (**I**) Western blotting for YAP and p-YAP after treatment with YAP inhibitors. The data are shown as the mean ± SEM, n = 3. *P < 0.05, **P < 0.01, ***P < 0.001

RT-qPCR and Western blotting results showed that miR-155-5p overexpression and Bmal1 inhibition decreased ALP activity (Fig. 2e), weakened the expression of osteogenic differentiation-related genes including Runx2, Ocn, and Alp, hindered the formation of mineralized nodules (Fig. 2h-j), and inhibited the expression of Ocn (Fig. 3a). On the contrary, miR-155-5p interference/Bmal1 overexpression positively regulated osteogenic differentiation. The above results revealed that miR-155-5p promoted the aging of mouse BMSCs and blocked osteogenic differentiation ability in vitro, which had the opposite effect to that of Bmal1.

### miR-155-5p and Bmal1 Regulated the Hippo Signaling Pathway in BMSCs

To explore the mechanism by which Bmal1 regulates BMSCs, the chromatin immunoprecipitation of the BMAL1 protein was conducted. GO enrichment analysis of peak genes showed that BMAL1 was correlated with cell differentiation and development in biological processes (Fig. 3b). The KEGG pathway analysis revealed that the Hippo pathway had a close relationship with Bmal1. Among the 154 genes related to the Hippo pathway, 103 had peaks (Fig. 3c, Supplementary Table 2). Figure 3d shows that the core components of the Hippo pathway, Mst1/2 and Lats1/2, were both peak genes. We downloaded the gene expression profiles from RNA-seq performed on BMSCs after knockdown of the Bmal1 gene from the GEO (Gene Expression Omnibus) database (ID: GSE208729) and performed differential analysis of RNA expression using DEseq2; the results showed a significant difference in the expression of Lats2 and Taz (*P* < 0.05) (Fig. 3e, Supplementary Table 3). Furthermore, RT-qPCR and Western blotting assays showed that overexpression of miR-155-5p/suppression of Bmal1 promoted the mRNA expression of Mst1, Mst2, Lats1, and Lats2 and the protein levels of Lats1, Lats2, p-MST1/2 and p-YAP, while also suppressing YAP and TAZ, resulting in a significantly higher p-YAP/YAP ratio (Fig. 3f, g). Moreover, miR-155-5p inhibition/Bmal1 overexpression both downregulated the mRNA expression of Mst1, Mst2, Lats1, and Lats2 and stimulated the mRNA and protein levels of YAP and TAZ while inhibiting the phosphorylation of MST1/2 and YAP, leading to a strongly diminished p-YAP/YAP ratio (*P* < 0.05). Both miR-155-5p and Bmal1 had no significant effect on the protein expression of MST1 and MST2.

**Fig. 4:**
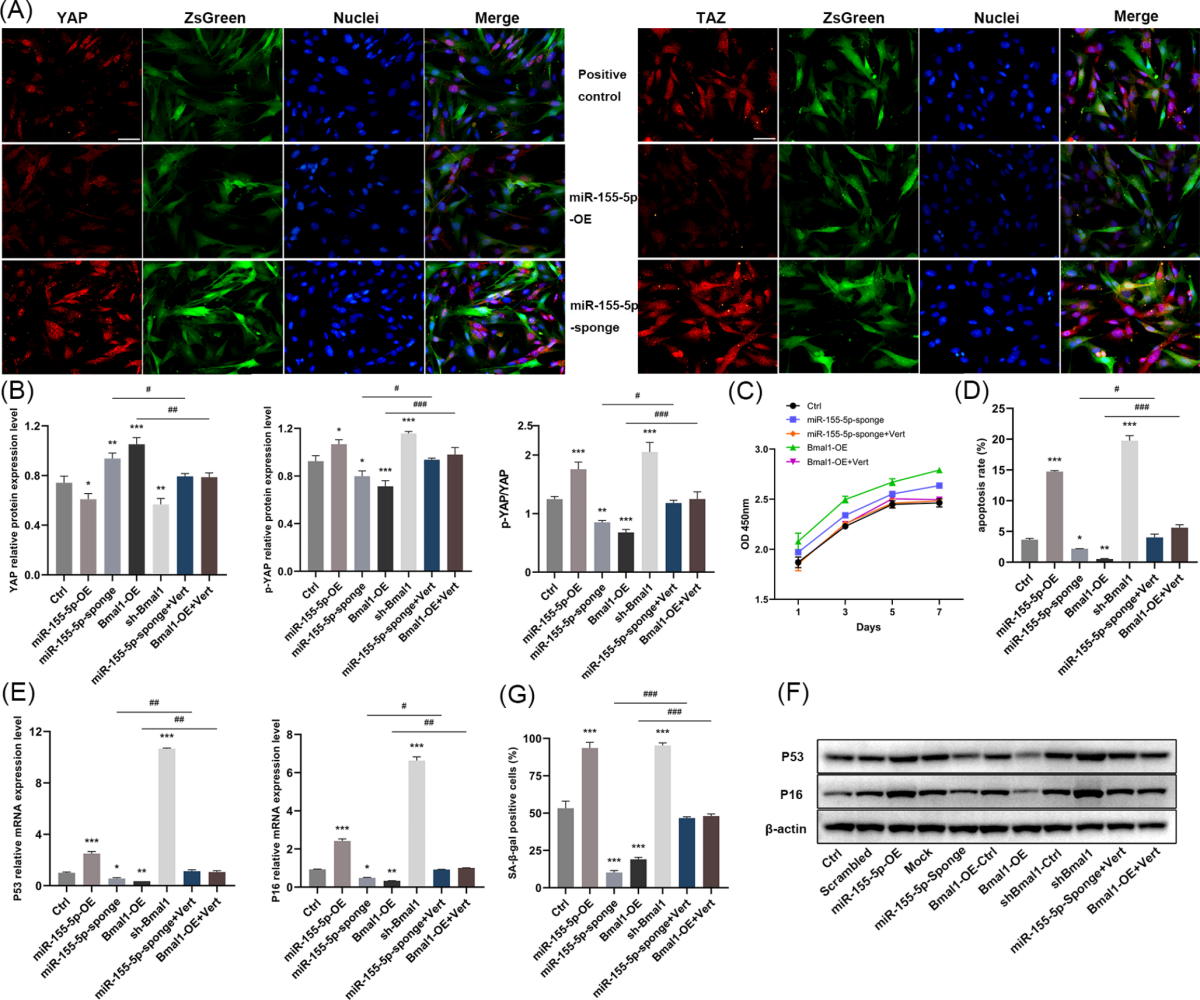
The YAP inhibitor eliminated the therapeutic effect of miR-155-5p inhibition and Bmal1 overexpression on the senescence of BMSCs. (**A**) Immunofluorescence staining for YAP and TAZ in BMSCs of miR-155-5p overexpression and miR-155-5p inhibition groups, scale bar = 50 μm. (**B**) Western blotting for YAP and p-YAP after treatment with YAP inhibitors and the ratio of p-YAP to YAP levels. (**C**) Proliferation of BMSCs was evaluated by CCK-8 assay. (**D**) The apoptosis ratio of BMSCs was detected by flow cytometry after treatment with the YAP inhibitor. (**E**) RT-qPCR analysis of P53 and P16. (**F**) Western blotting for P53 and P16. (**G**) Effect of YAP inhibitors on percentage of β-gal positive staining cells. The data are shown as the mean ± SD, n = 3. *P < 0.05, **P < 0.01, ***P < 0.001, ^#^*P* < 0.05, ^##^*P* < 0.01, ^###^*P* < 0.001

In line with those findings, the immunofluorescence staining assay indicated that the levels of YAP and TAZ in the miR-155-5p overexpression/Bmal1 inhibition group were lower than those in the control group, and their translocation into the nucleus was greatly blocked (Figs. 3h and 4a). However, the overall fluorescence intensity of YAP and TAZ was stronger in the miR-155-5p inhibition/Bmal1 overexpression groups, and the fluorescence in the nucleus was largely augmented. Accordingly, the above results demonstrated that miR-155-5p could activate the Hippo signaling pathway by inhibiting the expression and nuclear translocation of YAP and TAZ, while Bmal1 could suppress the Hippo pathway and stimulate the expression and translocation of YAP and TAZ.

### miR-155-5p and Bmal1 Played the Regulatory Roles in BMSCs Mainly through the Hippo Signaling Pathway

To further investigate whether the Hippo signaling pathway is the main pathway through which miR-155-5p and Bmal1 regulate the aging and osteogenic differentiation of BMSCs, the miR-155-5p inhibition and the Bmal1 overexpression groups were treated with verteporfin, the YAP inhibitors. The western blot analysis showed that YAP inhibitors successfully suppressed the protein level of YAP (Fig. 3i), promoted the phosphorylation of YAP, and distinctly reduced the p-YAP/YAP ratio (Fig. 4b).

**Fig. 5:**
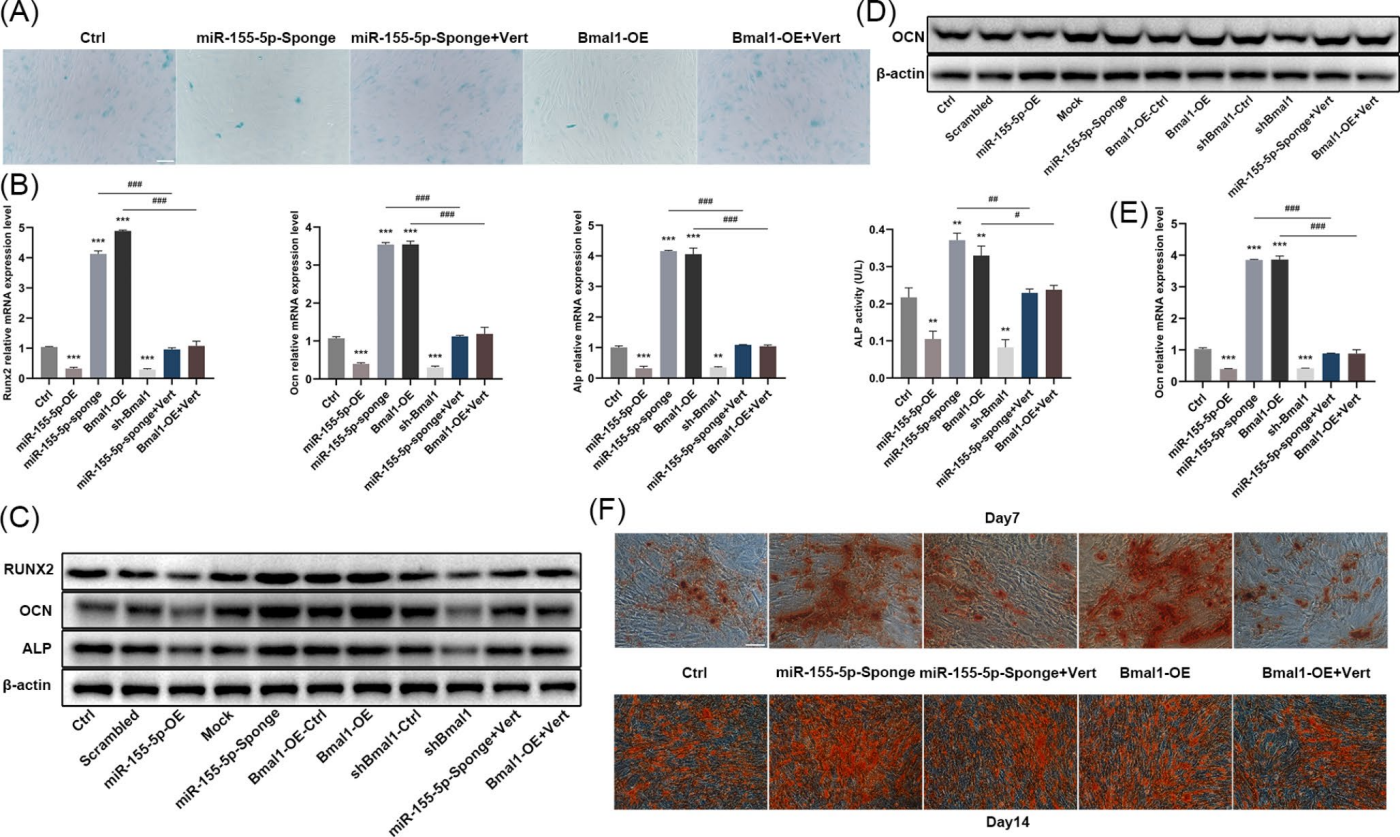
The YAP inhibitors eliminated the therapeutic effect of miR-155-5p inhibition and Bmal1 overexpression on the osteogenic differentiation ability of BMSCs. (**A**) Representative image of BMSCs stained by β-gal after treatment with YAP inhibitors, scale bar = 200 μm. (**B**) RT-qPCR analysis of Runx2, Ocn, Alp, and ALP activity was detected after 7 d of osteogenic induction in different groups. (**C**) Western blotting for RUNX2, OCN and ALP after 7 d of osteogenic induction. (**D**) Western blotting for OCN after 14 d of osteogenic induction. (**E**) RT-qPCR analysis of OCN after 14 d of osteogenic induction. (**F**) Alizarin red staining for mineralized nodules after 7 d and 14 d of osteogenic induction, scale bar = 200 μm. The data are shown as the mean ± SD, n = 3. **P < 0.01, ***P < 0.001, ^#^*P* < 0.05, ^###^*P* < 0.001

The results of age-related indicators in each group showed that verteporfin disrupted the therapeutic effects of miR-155-5p interference and Bmal1 overexpression on the proliferative capacity of BMSCs and abolished their inhibition on cell apoptosis (Fig. 4c-f). Beyond that, β-gal staining showed a robust enhancement in cell senescence (Fig. 5a). Additionally, verteporfin noticeably eliminated the upregulation by miR-155-5p interference and Bmal1 overexpression on the expression of Runx2, Ocn and Alp, also markedly diminished the activity of ALP and the formation of calcified nodules (Fig. 5b-f). Apparently, the results indicated that the YAP inhibitor abrogated the effects of miR-155-5p interference and Bmal1 overexpression on the amelioration of cellular senescence and the promotion of bone formation. Taken together, these data suggest that the Hippo pathway was the prime signaling pathway that involved in the modulation of aging and osteogenic differentiation of BMSCs by miR-155-5p and Bmal1.

## Discussion

Mammalian clock genes regulate biological rhythm by forming a self-regulating negative feedback network through the oscillator system. Emerging data indicate that the clock gene Bmal1 plays a crucial role in the aging process. Bmal1 knockout mice exhibited disturbed biological rhythms, accelerated aging, a shortened lifespan and a low bone mass phenotype, which were significantly correlated with bone aging [8, 28]. Overexpression of Bmal1 helped alleviate senescence by repairing DNA damage, restoring the osteogenic ability of BMSCs [13, 29]. However, the mechanisms upstream and downstream of Bmal1 in the regulation of BMSC aging are not well characterized. This study was aimed to explore the main mechanisms by which Bmal1 regulates the aging and osteogenic differentiation ability of BMSCs. We screened the candidate miRNAs that were most likely to target Bmal1 by searching the databases. The miR155 host gene is located in the exon part of B-cell integration cluster genes, in which miR-155-5p is encoded and was first found to play a specific role in the immunity, inflammation and tumorigenesis [30]. Some experimental studies have shown that miR-155-5p is also involved in the regulation of bone metabolism, affecting both osteogenic and osteoclastic activity [31, 32]. However, few studies have examined its roles in BMSCs. Here, our results clearly showed that the expression of Bmal1 in aged mice was lower than that in the young mice, which is in line with other findings [33], whereas miR-155-5p was higher in aged mice compared with that in the young group. Inhibition of miR-155-5p in aging BMSCs promoted the expression of Bmal1, while overexpression inhibited Bmal1. Thus, Bmal1 was speculated to be targeted by miR-155-5p. The results of the luciferase reporting assay suggested that miR-155-5p mimics could recognize the binding sites of Bmal1 and evidently reduce the relative fluorescence activity. In contrast, the relative luciferase activity did not change in the mutant group. Therefore, the decrease in Bmal1 expression with age may be mediated by miR-155-5p. Consistent with other studies, miR-155 was reported to bind to the 3’UTR of Bmal1 in macrophages to orchestrate the circadian clock and promote the inflammatory response; inhibition of miR-155 upregulated Bmal1 and alleviated brain trauma induced by cerebral hemorrhage [34].

However, the miRNA expression is tissue specific and can be regulated by some transcription factors. Moreover, ChIP-Seq for Bmal1 showed that there was an enrichment peak in the upstream of miR-155-5p. To further explore the relationship between Bmal1 and miR-155-5p in BMSCs, lentivirus transfection was used to study their mutual effects. Interestingly, we found that miR-155-5p suppressed Bmal1, and Bmal1 also had an inhibitory effect on miR-155-5p. The expression of miR-155-5p increased after downregulating Bmal1, and the difference was statistically significant (*P* < 0.05). The UCUS database was used to analyze the promoter sequence of miR-155, and the JASPAR database was used to predict the potential binding sites of BMAL1. The analysis results indicated that BMAL1, as a transcription factor, had a higher probability of downregulating the expression of miR-155-5p (Score: 5.39818). Additionally, previous studies have shown that Bmal1 can suppress miR-155 and LPS-induced proinflammatory responses in myeloid cells by regulating IL10 [35] and inhibit miR-155 by modulating P65 [36]. Further experiments are needed to verify the specific mechanism of miR-155-5p inhibition by Bmal1 in BMSCs. The core components of mammalian Clock genes, in addition to Bmal1, include Clock, Per2, Cry and Rev-erbα. They could form a self-regulating negative feedback network through an oscillator system, in which the BMAL1/CLOCK dimer could bind to the E-box elements of Per2, Cry and Rev-erbα to activate their transcription [37]. Therefore, we speculated that the suppression of Per2, Cry and Rev-erbα by miR-155-5p may be due to the chain reaction of the inhibition on the transcriptional promoter BMAL1.

Many studies have evidenced that Bmal1 plays a vital role in the maintenance of bone homeostasis. Overexpression of Bmal1 in MC3T3 cells promoted osteogenic differentiation, while the number of osteoblasts in Bmal1^−/−^ mice was reduced, showing a low bone mass phenotype, and gradually deteriorated with age [13]. Consistent with most of the present findings, we found that overexpression of Bmal1 promoted the proliferation of BMSCs in mice, elevated the levels of RUNX2, OCN, and ALP, enhanced the activity of ALP and the formation of mineralized nodules, and simultaneously inhibited cell apoptosis and senescence. Rather, there were studies suggested that knockdown of Bmal1 promotes bone formation [38], which may be related to the different Cre driving factors used in the studies, such as Osx-Cre, Col1a-Cre, and Prx1-Cre, and the different timing of gene knockdowns [39]. In contrast to Bmal1, miR-155-5p inhibited the proliferation and osteogenic differentiation of BMSCs, promoted cell apoptosis and accelerated senescence. These effects may be indirect due to the inhibition on Bmal1 by miR-155-5p. Similarly, previous literature has shown that miR-155 inhibits the migration of BMSCs [40], but there is a lack of reports on its effects on the proliferation and senescence of BMSCs. However, it is in keeping with other studies showing that miR-155-5p tends to inhibit osteogenesis. lncRNA MALAT1 was reported to promote osteogenic differentiation of human periodontal ligament stem cells by inhibiting miR-155-5p [41]. miR-155-5p inhibited the osteogenic differentiation and calcification of aortic smooth muscle cells and reduced vascular sclerosis [42]. This study shows for the first time the special role of miR-155-5p in the aging of BMSCs.

The mechanism by which Bmal1 regulates the aging and osteogenic differentiation of BMSCs remains controversial. It has been suggested that Bmal1 can regulate the proliferation and matrix mineralization of BMSCs through the BMP signaling pathway [12] or the bone homeostasis by the NF-κB pathway in type 2 diabetes mellitus [15]. However, it was not discussed in-depth whether these pathway act as prime pathways for Bmal1 to regulate BMSCs, and the regulatory mechanism is unclear. Moreover, Bmal1 was found to inhibit the activity of NF-κB pathway indirectly. Some studies have stated that Bmal1 promotes the osteogenic differentiation of BMSCs by reducing the expression of GSK-3β, subsequently activating the Wnt/β-catenin pathway or the P53 pathway. However, the inhibition of GSK-3β alone could not activate the Wnt pathway [14], while inhibition of P53 could only partially restore the osteogenic ability of BMSCs in patients with type 2 diabetes [15]. To identify the main core pathways involved in the regulation of BMSCs by Bmal1, we found significant enrichment in the Hippo pathway by ChIP-Seq and KEGG analysis. Mst1/2 and Lats1/2 were both peak genes. However, only Lats2 showed a significantly reduced RNA and protein expression level after suppressed the Bmal1 as evidenced by RNA-Seq database analysis and our results, which indicated that Lats2 was the most possible gene regulated by Bmal1 directly. The specific targeting relationship between them will be explored in our following research. Further results showed that Bmal1 could promote the expression and nuclear localization of YAP and TAZ, inhibit the phosphorylation of YAP, and subsequently inactivate the Hippo signaling pathway. As a mechanical signal transduction molecule, YAP plays a key role in bone remodeling and osteoblast differentiation regulated by mechanical stress and is selectively and highly expressed in osteoblast cell lines such as BMSCs and osteoblasts [43]. Recent studies have shown that YAP can promote osteogenic differentiation and cell proliferation, but inhibit lipogenic differentiation in mouse and human MSCs [44]. Likewise, Snail/Slug-YAP/TAZ axis was reported to upregulates self-renewal and osteogenic differentiation in skeletal stem cells, while nuclear YAP depletion by Verteporfin or RNAi induced the impaired proliferation and enhanced senescence in hMSCs [45, 46]. Few studies investigated the relationship between Bmal1 and Hippo signaling pathway. However, loss of Bmal1 was found to increase the intestinal tumor initiation by promoting the expression and activity of YAP, which was in contrary to our results [27]. To elucidate whether the Hippo pathway is the primary regulatory mechanism in the mouse BMSCs, YAP inhibitors was used to eliminate the high expression of YAP induced by Bmal1 overexpression and miR-155-5p inhibition, and the age-related phenotypes were compared. The in vitro experiments showed that verteporfin successfully regulated the Hippo signaling pathway and increased the p-YAP/YAP ratio. The inhibitors markedly reduced or even eliminated the therapeutic effect of Bmal1 overexpression/miR-155-5p inhibition on the proliferation and osteogenic differentiation of BMSCs and the amelioration of cell apoptosis and senescence. These findings indicated that the Hippo is the pivotal pathway in the regulation of BMSCs by Bmal1. Other critical signaling pathways reported to involve in the regulation of BMSC aging, such as Wnt/β-catenin and BMP2, interact with Hippo signaling pathway [47]. It was found that YAP maintains the nuclear levels of β-catenin and promoted the Wnt/β-catenin signaling-mediated osteogenesis. Overexpression of β-catenin in BMSCs along with knockdown of YAP partially rescued the osteogenic differentiation ability [43]. YAP and TAZ also promote the transcription of the target genes of TGF-β and BMP, while knockdown of YAP or TAZ leads to a reduction in BMP2-induced osteogenic differentiation [48]. In addition, YAP1 suppressed the osteoclasts differentiation and the activation of the NF-κB signaling [49]. A recent study showed that YAP/TAZ also impacts the Bmal1 and circadian rhythms in fibroblast, which might suggest a potential interaction relationship [50]. Above all, the Hippo pathway can not only regulate the transcription factors such as RUNX2 and PPARγ directly but also affect through the networks with other osteogenesis-related signaling pathways. However, the specific mechanism by which BMAL1 regulates the Hippo pathway in BMSCs needs further exploration.

miR-155-5p inhibited the expression and nuclear localization of YAP and TAZ, and activated the Hippo pathway concomitantly, which might be achieved by governing Bmal1. Recent studies also found that miR-155-5p could regulate other noncoding RNAs, such as miR-18a-5p or lncRNA Rncr2 to affect the Hippo pathway [51, 52]. The promoting effect of miR-155-5p inhibition on BMSC proliferation and osteogenic differentiation could be abolished by YAP inhibitors, suggesting that miR-155-5p regulates BMSC aging mainly through the Hippo pathway. Taken together, the above results indicated that the regulatory mechanisms upstream and downstream of Bmal1 in mouse BMSC aging and osteogenic differentiation have been demonstrated. Bmal1 could promote osteogenic differentiation and alleviate senescence of BMSCs by inhibiting the Hippo signaling pathway, and miR-155-5p could target Bmal1, thus blocking the osteogenic differentiation of BMSCs by activating the Hippo pathway. However, one miRNA may regulate multiple target genes. Can miR-155-5p act directly on the core components of the Hippo pathway? It is well known that one gene can be regulated by multiple miRNAs. Our previous study found that Bmal1 could also be regulated by miR-142-3p. Therefore, what is the possible relationship between other miRNAs and miR-155-5p? Who dominates the regulation of Bmal1? Further investigations are warranted to determine whether the miR-155-5p/Bmal1/Hippo pathway has the same impact in vivo.

## Conclusion

In summary, our results identified the primary mechanism by which Bmal1 regulates the aging and osteogenic differentiation of mouse BMSCs. miR-155-5p could target Bmal1 and had a mutual inhibitory relationship with Bmal1. Notably, miR-155-5p and Bmal1 regulate the aging and osteogenic differentiation of BMSCs mainly through the Hippo pathway, and YAP inhibitors could eliminate their effects on BMSC aging. Our findings shed new light on the mechanism of BMSCs regulated by Bmal1 and suggest novel therapeutic targets for aging-related osteoporosis. More studies are needed to validate the specific regulatory mechanism of Bmal1 on BMSCs in vivo.

### Electronic Supplementary Material

Below is the link to the electronic supplementary material.

Supplementary Material 1

Supplementary Material 2

## Data Availability

Department of Orthodontics, West China School & Hospital of Stomatology, Sichuan University, 14, 3Rd Section of Ren Min Nan Rd, Chengdu 610041 China.
