# Supplementary material for: miR-155-5p/Bmal1 Modulates the Senescence and Osteogenic Differentiation of Mouse BMSCs through the Hippo Signaling Pathway
Source: Stem Cell Rev Rep. 2023 Dec 27;20(2):554–67. doi: 10.1007/s12015-023-10666-3 (PMC10837250; doi:10.1007/s12015-023-10666-3)
Supplement: Supplementary file 1 — Supplementary Material 1 [file 12015_2023_10666_MOESM1_ESM.docx]

**Title:** miR-155-5p/Bmal1 modulates the senescence and osteogenic differentiation of mouse BMSCs through the Hippo signaling pathway

**Journal Name**：Stem cell reviews and reports

Authors：Lanxin Zhang^1^, Chengxiaoxue Zhang^1^, Jiawen Zheng^1^, Yuhong Wang^2^, Xiaoyu Wei^1^, Yuqing Yang^1^, Qing Zhao^1^

Department of Orthodontics, State Key Laboratory of Oral Disease & National Clinical Research Center for Oral Diseases, West China School & Hospital of Stomatology, Sichuan University, 14, 3Rd Section of Ren Min Nan Rd, Chengdu 610041 China.

Qing Zhao: fanfan_qing@163.com

The sequences of primer used are listed in Table1

| Primer name | Forward | Reverse |
| --- | --- | --- |
| miR-155-5p | 5’-GCGCGTTAATGCTAATTGTGAT-3’ | 5’-ACTGCAGGGTCCGAGGTATT-3’ |
| Bmal1 | 5’-CTCAGGGCAGCAGATGGATT-3’ | 5’-CGCAGTGTCCGAGGAAGATA-3’ |
| U6 | 5’-AGCACATATACTAAAATTGGAACGAT-3’ | 5’-ACTGCAGGGTCCGAGGTATT-3’ |
| β-actin | 5’-GCTGCGCTGGTCGTCG-3’ | 5’-GGCCTCGTCACCCACATAG-3’ |
| P53 | 5’-TTCTTCTTCCTACAGTACTA-3’ | 5’-AGTTGCAAACCAGACCTCAG-3’ |
| P16 | 5’-GGAAATTGGAAACTGGAAGG-3’ | 5’-CTGCCCATCATCATGACCTG-3’ |
| Alp | 5’-TCCTCTATGTAGGTTCCA-3’ | 5’-TTTGTCACTTGCTCTTTG-3’ |
| Runx2 | 5’-GCTTGATGACTCTAAACC-3’ | 5’-ACACCTACTCTCATACTG-3’ |
| Ocn | 5’-CAACATCTTCCGAATCAG-3’ | 5’-TGGACTTAGAGATAGCATAG-3’ |
| Mst1 | 5’-CCATCGGTACAAGCAATA-3’ | 5’-TACTTCGTGTTACATTCCT-3’ |
| Mst2 | 5’-TTGAACATAACAGCACCAT-3’ | 5’-CTTCCTCTTCCTCCTCTT- 3’ |
| Lats1 | 5’-CAAGGAAGATGATAGTGAGA-3’ | 5’-CGAACAGTGATAGGTGAA-3’ |
| Lats2 | 5’-AGAAGGAGTCTAACTACAAC-3’ | 5’-GATGCCTAGAGTCTTGATT-3’ |
| Yap | 5’-CAGTTACAGATGGAGAAG-3’ | 5’-GATTGATATTCCGTATTGC-3’ |
| Taz | 5’-AAAGGGATGGACTTCATT-3’ | 5’-CAGGAACTCAGAACTCAT-3’ |
| Clock | 5’-CGCAGTTAATGCTAATTGTGATAGG-3’ | 5’-AGTGCGTGTCGTGGAGTCG-3’ |
| Cry | 5’-TAGCCAGAATGACCTTATTG-3’ | 5’-AGTGTCCGAGGAAGATAG-3’ |
| Per2 | 5’-GGAAATTGGAAACTGGAAGG-3’ | 5’-CTGCCCATCATCATGACCTG-3’ |
| Rev-erbα | 5’-TTCTTCTTCCTACAGTACTA-3’ | 5’-AGTTGCAAACCAGACCTCAG-3’ |

The differentially expressed genes enriched in the Hippo pathway are presented in Table2

| Term | Genes |
| --- | --- |
| The Hippo signaling pathway | \| \| Actb \| \| --- \| \| Afp \| \| Birc5 \| \| Axin1 \| \| Axin2 \| \| Bmp5 \| \| Bmp6 \| \| Bmp8a \| \| Bmpr1b \| \| Bmpr2 \| \| Btrc \| \| Ctnna2 \| \| Ccnd1 \| \| Dlg1 \| \| Dlg4 \| \| Dvl1 \| \| Dvl3 \| \| Fzd1 \| \| Fzd4 \| \| Fzd6 \| \| Fzd7 \| \| Gdf5 \| \| Gli2 \| \| Id2 \| \| Itgb2 \| \| Lats1 \| \| Lef1 \| \| Smad1 \| \| Smad2 \| \| Smad3 \| \| Smad4 \| \| Smad7 \| \| Nf2 \| \| Serpine1 \| \| Ppp1ca \| \| Ppp2ca \| \| Tcf7 \| \| Tcf7l2 \| \| Tead1 \| \| Tead2 \| \| Tgfbr2 \| \| Wnt10a \| \| Wnt2 \| \| Wnt2b \| \| Wnt3 \| \| Wnt5a \| \| Wnt7b \| \| Wnt8b \| \| Ywhag \| \| Ywhah \| \| Dlg2 \| \| Amot \| \| Limd1 \| \| Lats2 \| \| Ppp2r2d \| \| Dlg3 \| \| Stk3 \| \| Gsk3b \| \| Ppp2r2b \| \| Pard6g \| \| Fzd10 \| \| Nkd1 \| \| Wtip \| \| Fbxw11 \| \| Csnk1d \| \| Bbc3 \| \| Gm4760 \| \| Gdf7 \| \| Crb2 \| \| Gdf6 \| \| Frmd6 \| \| Gm5110 \| \| Gm5279 \| \| Gm5388 \| \| Gm18429 \| \| Gm14609 \| \| Gm14824 \| \| Gm14981 \| \| Gm14655 \| \| Gm15158 \| \| Gm18443 \| \| Gm12805 \| \| Gm18484 \| \| Gm18489 \| \| Gm18700 \| \| \| --- \| --- \| --- \| --- \| --- \| --- \| --- \| --- \| --- \| --- \| --- \| --- \| --- \| --- \| --- \| --- \| --- \| --- \| --- \| --- \| --- \| --- \| --- \| --- \| --- \| --- \| --- \| --- \| --- \| --- \| --- \| --- \| --- \| --- \| --- \| --- \| --- \| --- \| --- \| --- \| --- \| --- \| --- \| --- \| --- \| --- \| --- \| --- \| --- \| --- \| --- \| --- \| --- \| --- \| --- \| --- \| --- \| --- \| --- \| --- \| --- \| --- \| --- \| --- \| --- \| --- \| --- \| --- \| --- \| --- \| --- \| --- \| --- \| --- \| --- \| --- \| --- \| --- \| --- \| --- \| --- \| --- \| --- \| --- \| --- \| --- \| |

The RNA expression profiles summarized from the GEO database (ID: GSE208729) are listed in Table 3

| Genes related with Hippo pathway | Counts-Sh-Bmal1 | | | Counts-Control | | |
| --- | --- | --- | --- | --- | --- | --- |
|  | Group1 | Group2 | Group3 | Group1 | Group2 | Group3 |
| MST1 | 79 | 64 | 67 | 46 | 62 | 77 |
| LATS2 | 1618 | 1909 | 1869 | 1004 | 1175 | 1470 |
| LATS1 | 733 | 818 | 774 | 689 | 784 | 718 |
| YAP1 | 3538 | 3418 | 3675 | 2965 | 3280 | 3410 |
| TAZ | 131 | 114 | 89 | 260 | 248 | 246 |
| TEAD1 | 6937 | 7366 | 5717 | 5851 | 6993 | 6257 |
| TEAD2 | 984 | 826 | 890 | 458 | 378 | 431 |
| TEAD3 | 1160 | 1111 | 1076 | 1118 | 1013 | 1010 |
| TEAD4 | 199 | 196 | 211 | 172 | 172 | 136 |
| FRMD1 | 1 | 0 | 0 | 0 | 0 | 0 |
| MOB2 | 231 | 218 | 239 | 271 | 226 | 227 |
| SAV1 | 557 | 522 | 695 | 504 | 540 | 624.98 |
