# Supplementary material for: miR-155-5p/Bmal1 Modulates the Senescence and Osteogenic Differentiation of Mouse BMSCs through the Hippo Signaling Pathway
Source: Stem Cell Rev Rep. 2023 Dec 27;20(2):554–67. doi: 10.1007/s12015-023-10666-3 (PMC10837250; doi:10.1007/s12015-023-10666-3)

**Title:** miR-155-5p/Bmal1 modulates the senescence and osteogenic differentiation of mouse BMSCs through the Hippo signaling pathway

**Journal Name**：Stem cell reviews and reports

Authors：Lanxin Zhang^1^, Chengxiaoxue Zhang^1^, Jiawen Zheng^1^, Yuhong Wang^2^, Xiaoyu Wei^1^, Yuqing Yang^1^, Qing Zhao^1^

Department of Orthodontics, State Key Laboratory of Oral Disease & National Clinical Research Center for Oral Diseases, West China School & Hospital of Stomatology, Sichuan University, 14, 3Rd Section of Ren Min Nan Rd, Chengdu 610041 China.

Qing Zhao: fanfan_qing@163.com

**Supplementary** **Fig. S** Characterization of BMSCs and validation after virus transfection. (A) The levels of CD29 and CD34 were detected by flow cytometry. (B) Alizarin red staining of BMSCs after 21 d of osteogenic induction showed the formation of mineralized nodules, scale bar = 200 µm; Oil Red O staining of BMSCs after 14 d of adipogenic induction showed the formation of lipid droplets, scale bar = 100 µm. (C) mRNA levels were verified by RT-qPCR after overexpressing miR-155-5p in the young group and blocking miR-155-5p expression in the aged group. (D) Effects of miR-155-5p on the expression level of BMAL1 was detected by Western blotting. (E) The potential binding sites on Bmal1 by miR-155-5p as predicted by TargetScan and miRanda databases. (F) RT-qPCR analysis of Bmal1 and miR-155-5p in BMSCs after transfection with lentivirus. (G) Western blotting for Bmal1 after transfection with lentivirus. The data are shown as the mean±SD, n=3. **P<0.01, ***P<0.001

**Fig. S**


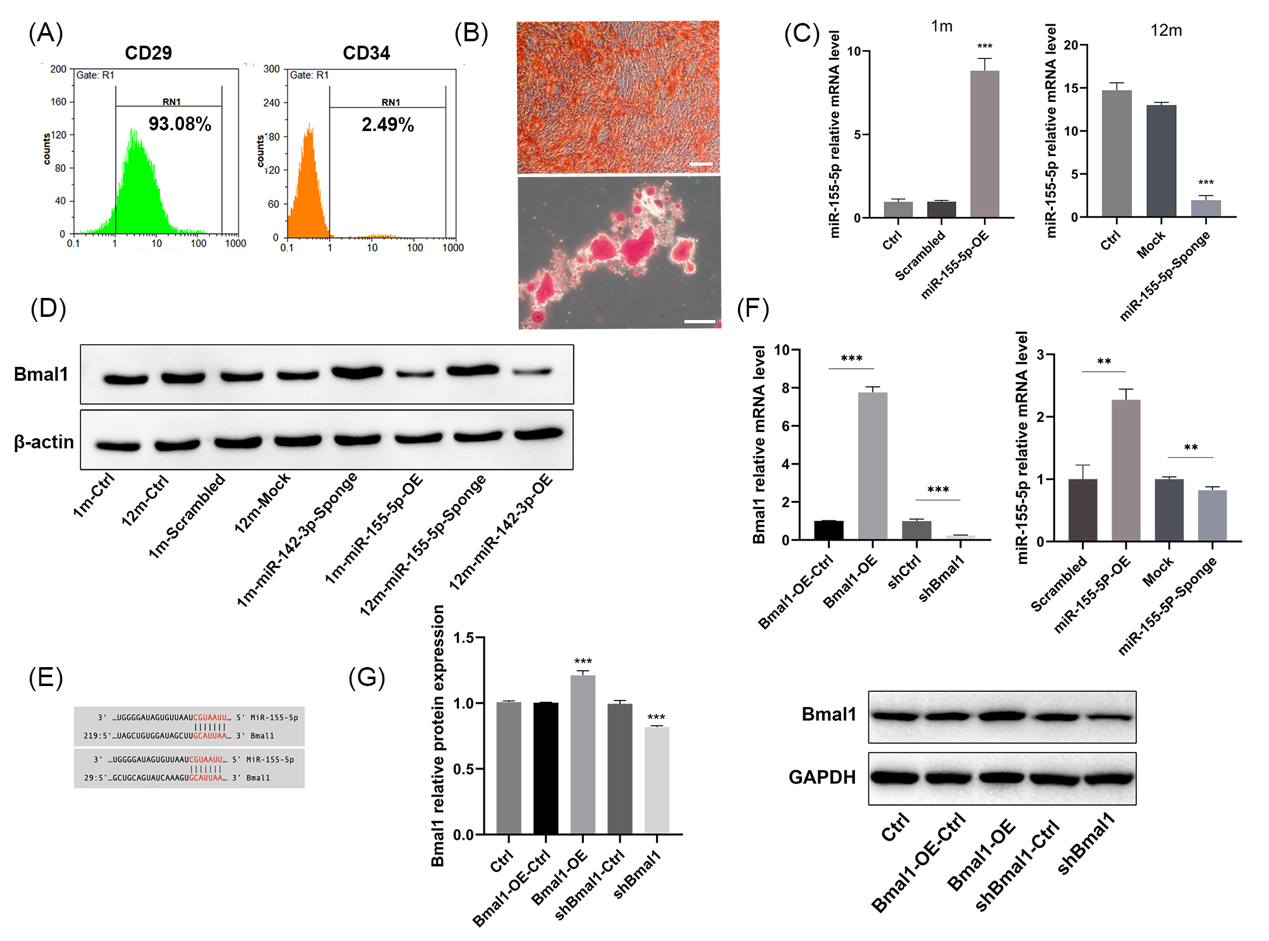

Supplement: Supplementary file 2 — Supplementary Material 2 [file 12015_2023_10666_MOESM2_ESM.docx]
